# High‐Performance Flexible 2D Tellurium Semiconductor Grown by Isolated Plasma Soft Deposition for Wearable and Flexible Temperature Sensors

**DOI:** 10.1002/smtd.202500379

**Published:** 2025-06-29

**Authors:** Tae‐Yang Choi, Jun‐Hyeok Kang, Jong‐Hyun Jang, Han‐Ki Kim

**Affiliations:** ^1^ School of Advanced Materials Science & Engineering Sungkyunkwan University (SKKU) 2066 Seobu‐ro, Jangan‐gu Suwon Gyeonggi‐do 16419 Republic of Korea

**Keywords:** 2D semiconductor, isolated plasma soft deposition, large‐area, tellurium, wearable temperature sensor

## Abstract

High‐quality flexible 2D tellurium (Te) semiconductors on a six‐inch Si wafer and polyethylene terephthalate substrate using the isolated plasma soft deposition (IPSD) technique are successfully fabricated. Unlike conventional sputtering systems, the IPSD process minimizes direct plasma irradiation and plasma damage, thereby preserving the unique helical chain structure of the 2D Te layer. The integration of oxygen plasma treatment and in situ substrate heating significantly enhanced both the adhesion and crystallinity of the 2D Te layer. The optimized 2D Te layer exhibited exceptional properties, including a high carrier mobility of 103 cm^2^ V^−1^ s^−1^, a smooth surface roughness of 0.778 nm, and a critical bending radius of 12 mm. When integrated into temperature sensors, the 2D Te/PET demonstrated high sensitivity, exhibiting a negative temperature coefficient response across the 20–40 °C range. Moreover, the IPSD‐grown 2D Te layer demonstrated outstanding mechanical flexibility, with minimal resistance changes (<4%) during both bending and rolling tests. Long‐term stability assessments conducted over 100 days revealed resistance variations of less than 1%, highlighting the material's robust reliability. These findings position the IPSD process as a promising physical vapor deposition technique for scalable fabrication of large‐area 2D Te layers, enabling their integration into wearable and flexible electronic devices.

## Graphical abstract

A high‐performance flexible 2D Te semiconductor is fabricated using IPSD, preserving its helical chain structure while enhancing crystallinity. The optimized Te layer exhibited high mobility (103 cm^2^ V^−1^ s^−1^), smooth surface roughness (0.778 nm), and a stable negative temperature coefficient response (20–40 °C). The IPSD‐grown Te film maintained exceptional uniformity across a large area, while the Te/PET sensor showed high reliability with minimal resistance changes (<4%) under repeated bending and rolling.

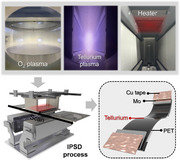

## 1 Introduction

Sensor technologies play a pivotal role in modern systems by facilitating rapid data collection for real‐time monitoring and prediction, which are crucial in the era of smart technologies.^[^ ^1^, ^2^, ^3^ ^]^ The scope of sensor applications is continuously expanding, now including pressure, image, and gas sensors, with an increasing range of potential uses.^[^ ^4^, ^5^, ^6^ ^]^ Among the various sensor types, temperature sensors are particularly important due to their wide applicability, from industrial environments to medical and healthcare settings. In particular, temperature sensors are critical for medical monitoring, where the demand for high‐performance temperature sensors capable of detecting changes in the human body is growing.^[^ ^7^, ^8^ ^]^ Body temperature is a key indicator for assessing health and physical conditions, enabling the diagnosis of diseases, stress level analysis, and other evaluations through temperature fluctuations. Consequently, high‐sensitivity temperature sensors are essential for real‐time health monitoring, supporting the advancement of personalized healthcare. However, unlike static environments, the human body experiences various movements, including acrobatic ones, necessitating the development of more flexible devices.^[^ ^9^, ^10^ ^]^ To meet these requirements, organic and amorphous semiconductor materials are actively researched and employed in the fabrication of flexible sensors.^[^ ^11^, ^12^, ^13^ ^]^ Recently, 2D materials have emerged as promising candidates for flexible devices due to their atomic‐scale thinness, offering exceptional flexibility and mechanical stability that make them well‐suited to the body's diverse range of movements.^[^ ^14^, ^15^ ^]^ Unlike bulk materials, which have a low surface‐to‐volume ratio and limited responsiveness of electrical signals to environmental variations, 2D materials, exhibit unique and advantageous properties despite, their thin‐layered structure. Their intralayer covalent bonds, coupled with weak van der Waals interactions between layers, allow for the formation of atomically thin yet stable layers, resulting in high carrier mobility and excellent electrical performance.^[^ ^16^, ^17^, ^18^, ^19^ ^]^ Consequently, 2D materials offer rapid responsiveness to environmental variations, along with superior flexibility and mechanical stability, which enhances their suitability for wearable temperature sensors. Among various 2D materials, tellurium (Te) has recently been recognized for its distinctive characteristics, including piezoelectric and thermoelectric performance as well as tunable direct bandgap.^[^ ^20^, ^21^ ^]^ Due to its unique helical chain structure, Te exhibits exceptional flexibility, allowing its thin films to withstand diverse physical deformations, thereby enhancing its potential for close‐contact applications, such as wearable temperature sensors.^[^ ^22^, ^23^, ^24^ ^]^ With excellent carrier mobility exceeding 100 cm^2^ V^−1^ s^−1^ and strong resistance to oxidation, 2D Te maintains long‐term stability, unlike 2D transition metal dichalcogenides.^[^ ^25^, ^26^ ^]^ Moreover, the 2D Te layer can be fabricated at low processing temperatures, enabling its direct integration onto flexible substrates.^[^ ^27^, ^28^ ^]^ These attributes make 2D Te an ideal material for highly sensitive wearable temperature sensors to monitor the human body. Despite these advantages, research on the 2D Te layer remains limited compared to other 2D materials, leading to interest in its synthesis and application. Typically, 2D semiconductors such as MoS_2_ and WS_2_ are composed of binary elements, necessitating precise control over stoichiometry. In contrast, 2D Te, being a single‐element material, does not have this requirement, providing significant advantages for film deposition processes. This unique aspect of Te facilitates a broader range of synthesis approaches, further supporting its potential for practical application in flexible and wearable sensors. To achieve a uniform 2D Te layer, various methods such as chemical vapor deposition (CVD), thermal evaporation, and hydrothermal synthesis, have traditionally been employed.^[^ ^29^, ^30^, ^31^, ^32^ ^]^ However, each method has notable limitations that impede practical applications. CVD, while capable of producing high‐purity 2D Te, requires elevated processing temperatures, which can compromise thermal stability and potentially damage sensitive 2D materials. Thermal evaporation, although effective, often faces challenges in achieving consistent thickness and efficient, material usage. Furthermore, the very low processing temperatures (−80 °C) associated with thermal evaporation can negatively affect the physical stability of the Te film.^[^ ^33^, ^34^ ^]^ Hydrothermal synthesis offers a low‐temperature, energy‐efficient alternative but is hindered by challenges in achieving uniform thin films over large areas, thus limiting its scalability. To overcome the constraints of conventional methods, recent research has shifted toward employing utilizing sputtering techniques for 2D Te thin film deposition, which offer high deposition rates and exceptional thickness uniformity. A comparison of sputtering parameters, film thicknesses, and annealing conditions for Te thin film deposition is summarized in **Table** **1**.^[^ ^35^, ^36^, ^37^, ^38^, ^39^, ^40^, ^41^ ^]^ However, conventional sputtering processes are typically conducted at low temperatures, providing insufficient energy for the migration of deposited atoms. This limitation increases the likelihood of forming amorphous structures, particularly during the short deposition times required for ultrathin film fabrication. Although post‐treatment methods have been employed to mitigate these issues, they often introduce risks of oxidation and contamination, necessitating additional steps such as the deposition of passivation layers to protect the material.^[^ ^42^ ^]^ Additionally, in conventional sputtering systems, plasma directly interacts with the substrate, leading to re‐sputtering effects that hinder the uniform deposition of thin films over large areas. The ability to protect large‐area thin films is crucial for the mass production of Te, highlighting the need for alternative deposition methods that prevent direct plasma‐substrate interaction while ensuring consistent film quality, uniformity, and scalability.^[^ ^43^ ^]^

**Table 1:** Comparison of key parameters and methods in the fabrication of sputtered Te thin films for various applications.

No	Fabrication method	Substrate	Substrate size	Sputtering power	Sputtering pressure	Annealing temperature	Thickness	Application	Refs.
1	Reactive magnetron sputtering	SiO_2_/Si	4‐inch	20 W	2 mTorr	Post‐annealing 150 °C	10 nm	Transistor	[35]
2	Magnetron sputtering	PET	2‐inch	10 W	1.0 Pa	Room temperature	–	Photodetector	[36]
3	Reactive magnetron sputtering	SiO_2_/Si	–	–	–	Post‐annealing 150 °C	4 nm	Transistor	[37]
4	DC magnetron sputtering	SiO_2_/Si	–	20 W	2 mTorr	Post‐annealing 150 °C	4 nm	Transistor	[38]
5	DC magnetron sputtering	SiO_2_/Si	–	20 W	2 mTorr	Postannealing 150 °C	7 nm	Transistor	[39]
6	RF sputtering	SiO_2_/Si, PEN	–	6 W	2 mTorr	Room temperature	3–28 nm	Transistor, flexible OLED	[40]
7	RF sputtering, DC sputtering	Glass, Alumina substrate	–	100 W	0.67 Pa	In situ annealing 140 °C	100 nm, 300 nm	NO_2_ gas sensor	[41]
8	IPSD sputtering system	SiO_2_/Si, PET	6‐inch	50 W	2 mTorr	In situ annealing 50 °C	5–15 nm	Temperature sensor	This work

In this study, we address the limitations of conventional magnetron sputtering methods by utilizing isolated plasma soft deposition (IPSD) technology to fabricate a uniform 2D Te layer. The IPSD system isolates high‐energy plasma by positioning two targets opposite to each other, creating a high‐density plasma region that minimizes plasma‐induced damage to the 2D Te layer, thereby preserving its intrinsic physical and electrical properties.^[^ ^44^, ^45^, ^46^ ^]^ The scan‐based deposition technique further facilitates the formation of uniform, large‐area 2D Te, with coverage extending up to 6 inches. To achieve a high‐quality 2D Te layer on Si and PET substrate, we optimized the IPSD parameters, including O_2_ plasma pre‐treatment conditions and scan speed, resulting in a 2D Te layer with enhanced properties. The IPSD, integrated with an in situ annealing system, enables the fabrication of highly reproducible 2D Te layers while minimizing the risks of oxidation and contamination. Additionally, we evaluated the mechanical flexibility of the 2D Te layer grown by IPSD on a PET substrate for flexible temperature sensors. The 2D Te‐based temperature sensor demonstrated excellent high‐temperature sensitivity within the 20–40 °C range, which is typical for human body temperatures, thereby confirming its potential for real‐time physiological monitoring. Long‐term stability tests further confirmed the reliability of the 2D Te‐based sensors, underscoring their potential for high‐performance flexible electronics. These results provide a solid foundation for the integration of 2D Te layers into next‐generation sensor technologies, establishing 2D Te as a promising material for flexible, high‐sensitivity electronic devices.

## 2 Results and Discussion

**Figure 1:**
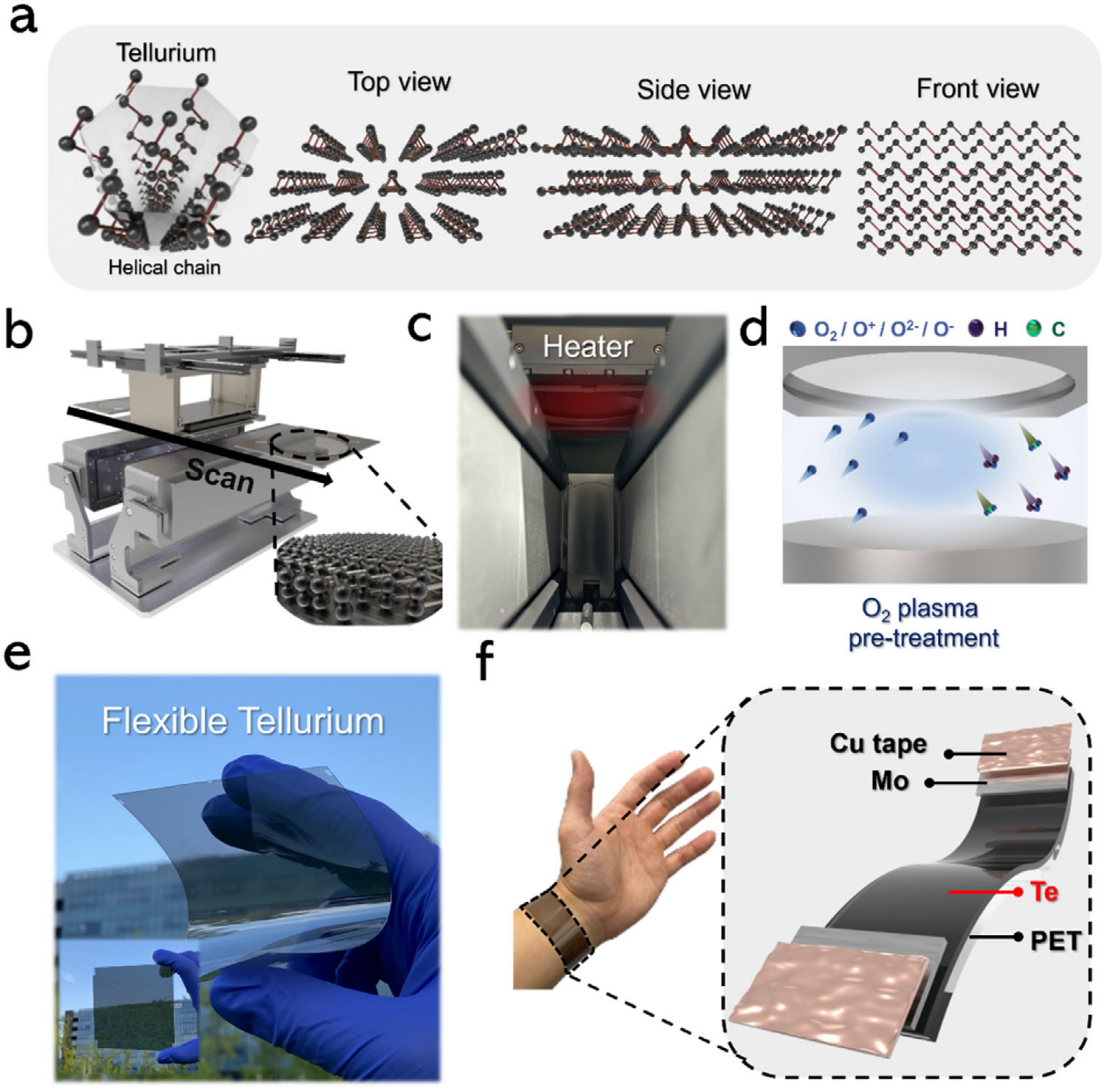
a) Atomic structure of 2D Te: helical chain and multi‐angle views. (top, side, front) b) Schematic of the isolated plasma soft deposition (IPSD) system and scanning process for uniform 2D Te layer. c) Image of substrate heater integrated into the IPSD system. d) Schematic of O_2_ plasma pre‐treatment for enhanced surface adhesion and cleanliness of Si and PET substrate. e) Actual images of large‐area 2D Te deposited on flexible PET substrate. f) Assembly of wearable 2D Te‐based temperature sensor on PET substrate.

The unique helical structure of 2D Te, which underpins its thermoelectric, piezoelectric, and optical properties, is illustrated in **Figure** **1**a. In this arrangement Te atoms form a helical pattern, resulting in 1D atomic chains that are interconnected by van der Waals forces. Each Te atom covalently bonds with only its two neighboring atoms, contributing to a distinctive 2D crystal structure. Unlike traditional 2D semiconductors, which possess strong interlayer bonding, this unique structure of 2D Te imparts high anisotropy and chirality. This gives rise to Te broad‐spectrum optical absorption, along with its inherent thermoelectric and piezoelectric properties. The helical architecture of 2D Te, therefore, suggests strong potential for a wide range of applications in optoelectronic and thermoelectric devices. However, preserving the unique helical chain structure while achieving uniform deposition of large‐area Te thin films remains a significant challenge while using conventional sputtering methods.

To address this challenge, the IPSD process was employed, enabling the uniform deposition of the 2D Te layer without compromising the integrity of the helical chain structure, as illustrated in Figure 1b. Additionally, Figure S1a (Supporting Information) shows a photograph of the IPSD system, while Figure S1b,c (Supporting Information) present images of high‐density plasma confined between Te targets in the IPSD process and O₂ plasma in pretreatment system before the deposition of the 2D Te layer. Furthermore, the linear substrate scan system of the IPSD, as demonstrated in Movie S1 (Supporting Information), allows for the deposition of uniform and large area 2D Te. The IPSD process effectively utilizes confined plasma generated by two Te targets facing each other, preventing direct plasma‐substrate interaction during deposition. This approach mitigates issues such as film non‐uniformity and plasma damage that can occur when plasma directly interacts with the film. Notably, the high kinetic energy (10–100 eV) of energetic particles generated by the plasma can lead to re‐sputtering of the 2D Te layer. This is particularly problematic for 2D semiconductor materials, which are highly sensitive to surface defects, especially when reduced to the ultra‐thin thicknesses necessary for optimal performance.^[^ ^47^, ^48^ ^]^ In the IPSD process, the substrate is placed outside the isolated plasma region, allowing the 2D Te layer to deposit with low kinetic energy similar to thermally vaporized atoms. Additionally, a substrate heater integrated above the substrate zig enables in situ annealing for crystallization, eliminating the need for additional thermal treatments as shown in Figure 1c.^[^ ^49^ ^]^ The substrate scanning system facilitates the formation of large‐area 2D Te thin films, demonstrating strong potential for scalable industrial applications. In IPSD system, O_2_ plasma pre‐treatment is applied in the load lock chamber before transferring the substrate to the main chamber, with the aim of improving the adhesion of the 2D Te layer, as shown in Figure 1d. During the pre‐treatment process, reactive oxygen species in the O_2_ plasma interact with organic contaminants and impurities on the substrate surface, generating hydrophilic polar groups. This increases the surface energy and reduces the contact angle, significantly improving adhesion during the 2D Te deposition process.^[^ ^50^, ^51^, ^52^ ^]^ Figure 1e demonstrates a large‐area flexible 2D Te layer deposited on PET substrate with the dimensions of 10 cm × 10 cm fabricated using the IPSD technique for wearable temperature sensors. Additionally, Figure 1f illustrates the structure of a 2D Te‐based wearable temperature sensor, incorporating Mo electrodes and Cu tape. Mo was selected as the electrode material considering its ability to form a low interfacial resistance contact with 2D Te, which is important for maintaining stable and accurate sensor operation. Moreover, Mo exhibits excellent thermal and chemical stability during device fabrication and shows superior chemical inertness compared to metals such as Ag or Cu that are prone to oxidation or reaction with Te, ensuring the long‐term reliability of the device.^[^ ^53^, ^54^ ^]^ To ensure the uniformity and quality of the 2D Te layer, a preliminary experiment was conducted to explore the effects of key process parameters—power, working pressure, and argon gas flow—on the growth rate and electrical characteristics of the 2D Te layers. Figure S2 (Supporting Information) presents the deposition rates of 2D Te layers as a function of power, working pressure, and gas flow, with detailed data provided in Table S2 (Supporting Information) to comprehensively present the effects of these process parameters. Figure S3 (Supporting Information) presents actual photographs of plasma under various process conditions, including power, working pressure, and gas flow. As the power increases, a noticeable enhancement in plasma density is observed, indicating higher ionization levels. However, while small variations in plasma density occur with changes in working pressure and gas flow, the differences are not significant to the naked eye. To detect subtle changes, Raman spectroscopy and Hall measurements were conducted as a function of power, working pressure, and gas flow, as shown in Figure S4 (Supporting Information). Based on the preliminary experiment, the optimal IPSD condition for the 2D Te layer was determined as a pulsed DC power of 50 W, working pressure of 2.0 mTorr, and Ar flow rate of 40 sccm.

**Figure 2:**
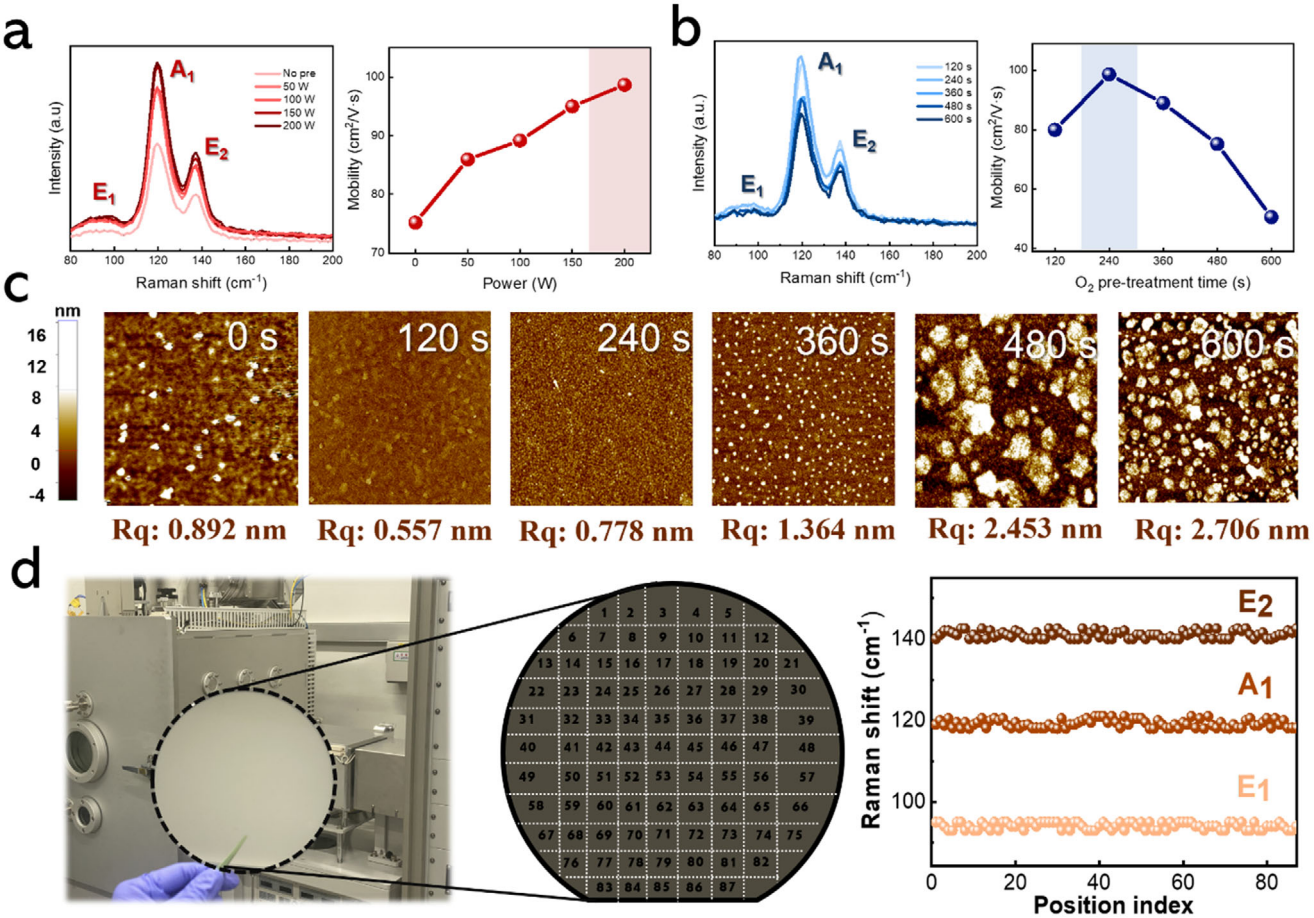
Raman spectroscopy and carrier mobility of the 2D Te layer with varying a) RF power and b) exposure time. c) Surface AFM images of the 2D Te layer with increasing oxygen plasma exposure time. d) Actual photograph of the 6‐inch Te layer on Si wafer with Raman mapping across 87 measurement points.

**Figure** **2a,b** depict the Raman spectroscopy analysis of a 2D Te layer deposited on a pretreated Si substrate, evaluated as a function of plasma power and exposure time. The results indicate that increasing the RF power during pretreatment significantly enhances the Raman peak intensity and carrier mobility of the 2D Te layer. All films compared in Figure 2a were deposited with a similar thickness of ≈10 nm, ensuring that the observed enhancement in Raman intensity reflects improved crystallinity rather than differences in material quantity. This improvement in crystallinity is attributed to the higher plasma energy reducing defect density and promoting the formation of larger grains. The observed correlation between enhanced Raman peak intensity and increased carrier mobility underscores the crucial of crystallinity in determining the quality of the 2D Te layer. Notably, optimal RF power for pretreatment was determined to be 200 W to avoid potential substrate warpage caused by excessive plasma energy. In contrast to RF power, the effect of oxygen plasma treatment duration on Raman intensity exhibited a non‐linear trend, as illustrated in Figure 2b. The highest Raman intensity was recorded at an exposure time of 240 s, after which a gradual decline was observed with further extended exposure. This behavior is attributed to a saturation effect in surface reactivity. Initially, oxygen plasma effectively removes contaminants and activates the surface. However, prolonged exposure results in the formation of an excessive oxide layer formation, which reduces surface reactively and negatively impacts the enhancement effect.

The mobility of the 2D Te layer peaked at 103 cm^2^ V^−1^ s^−1^ with an exposure time of 240 s and decreased with further increases in exposure time. Accordingly, the optimal oxygen pretreatment conditions were determined to be an RF power of 50 W and an exposure time of 240 s. The influence of exposure time on the surface morphology of the 2D Te layer was further analyzed using AFM, as depicted in Figure 2c. For consistency across all samples, all 2D Te layers were deposited to a uniform thickness of 15 nm. Initially, the surface roughness (Rq) of the untreated Te film was measured at 0.892 nm. After a 120 s exposure, the Rq value of the 2D Te layer significantly decreased to 0.557 nm, indicating improved surface uniformity. In contrast to the untreated Si substrate, the Te layer on the oxygen plasma‐treated Si wafer exhibited a more flattened surface, attributed to the hydrophilic properties of treated the substrate.^[^ ^47^ ^]^ At 240 s of exposure, AFM analysis showed a slight increase in surface roughness (Rq) to 0.778 nm, while maintaining a well‐developed defined 2D morphology of the Te layer, free from surface defects.^[^ ^47^, ^48^ ^]^ However, when the exposure time exceeded of 240 s, a significant increase in surface roughness was observed, accompanied by agglomeration of the Te layer. This agglomeration negatively impacted the uniformity of the Te layer, thereby degrading its electrical and optical properties. In particular, the increase in surface roughness introduced additional scattering centers for carriers, which could reduce effective mobility, a phenomenon especially critical in 2D materials where charge transport occurs near the surface. Although the overall roughness remained relatively low, the slight increase observed at longer exposure times may have contributed to the moderate mobility values measured. To evaluate the uniformity of the 2D Te layer grown by IPSD over a large‐area 6‐inch Si wafer, the wafer was segmented into 87 sections for detailed Raman mapping analysis as shown in Figure 2d.^[^ ^55^, ^56^ ^]^ The 2D Te layer on a 6‐inch Si substrate was fabricated by integrating the optimized pretreatment process with the substrate scanning system, as depicted in Figure S6 (Supporting Information). The 6‐inch wafer was divided into nine regions for deposition, using a scan speed of 2.0 m m^−1^, and the thickness distribution across these regions was analyzed, as shown in Figure S6 (Supporting Information). During each scan, the deposition occurred in a bidirectional manner (two passes per scan), allowing for precise control over the 2D Te growth. This method enabled us to optimize the scan speed to 2.0 m m^−1^, achieving a single‐layer deposition for every 0.5 scan. Additionally, the thickness uniformity of the 2D Te layer across the 6‐inch substrate was evaluated by analyzing on the deposition rates at various points, as shown in Figure S7 (Supporting Information). The error rate was quantified using the formula provided in the Supporting Information. The mapping results consistently revealed Raman peaks corresponding to the E_1_ mode, A_1_ mode, and E_2_ mode across all sampled locations, with a deviation of less than 3%. This minimal deviation highlights the excellent uniformity of the 2D Te layer deposited via the IPSD process. The precise control over deposition conditions enabled by the IPSD process ensures consistent crystallinity and uniformity while minimizing local defects across the entire wafer. This uniformity is crucial for high‐performance wearable sensor applications, where reliable operations depend on consistent electrical properties.

**Figure 3:**
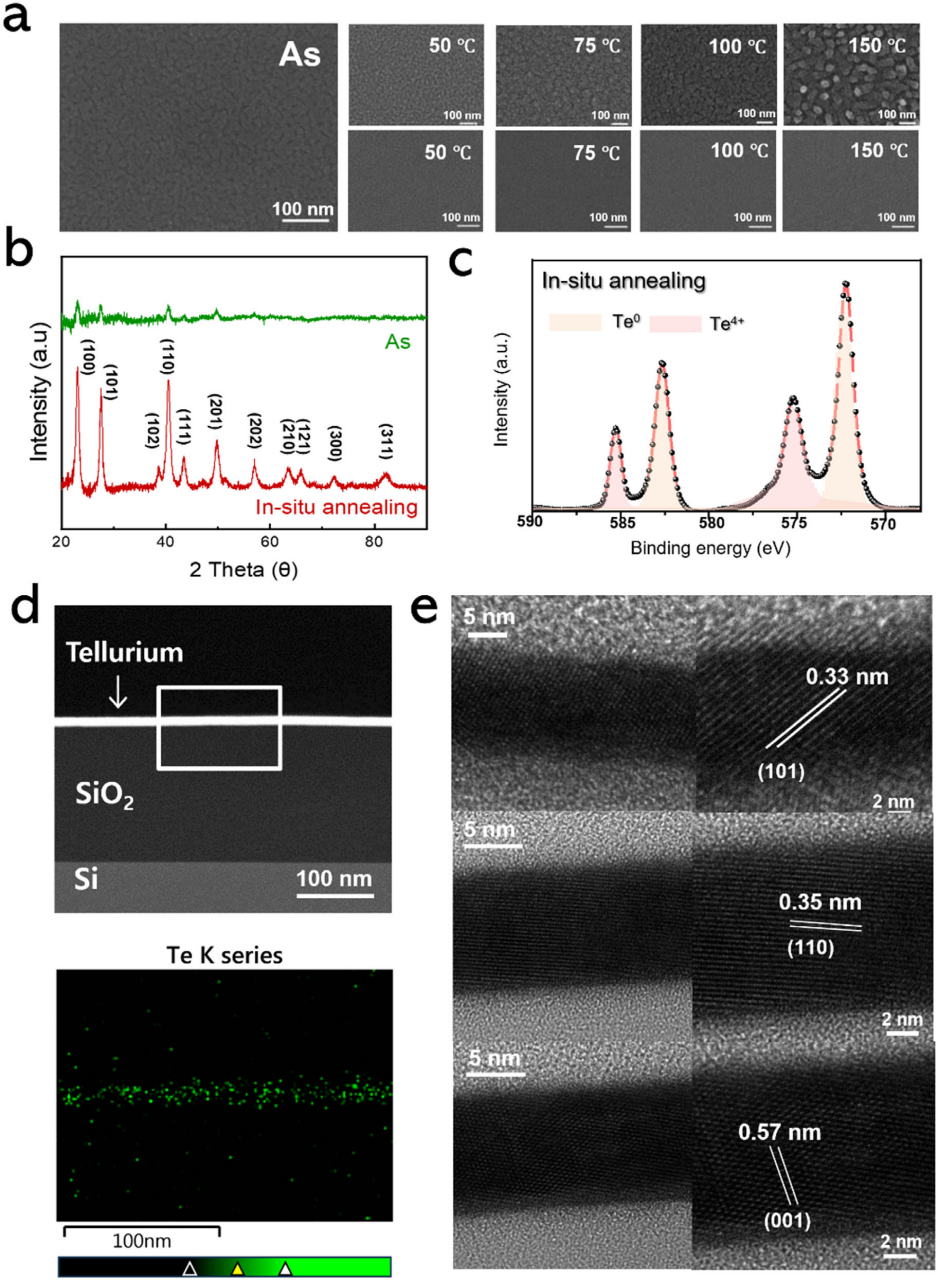
a) Surface FESEM images of IPSD‐grown Te layer under different annealing methods of RTA (upper) and in situ annealing (lower). b) XPS spectra of Te 3*d* peak for in situ annealed Te layer. c) XRD pattern comparing as‐deposited and in situ annealed Te layer at 50 °C. d) Cross‐sectional TEM image and EDS mapping of the IPSD‐grown Te layer. e) HRTEM images and enlarged view of the IPSD‐grown Te layer under in situ annealing, showing preferred (101), (110), and (001) planes.

**Figure** **3**a shows surface SEM images of the 2D Te layer processed using two different annealing methods for crystallization: rapid thermal annealing (RTA) and in situ annealing. The leftmost image depicts the as‐deposited Te layer without the annealing process, which exhibits a fine‐grained and smooth surface morphology. The upper row of SEM images in Figure 3a illustrates the IPSD‐grown Te layer, annealed by the RTA process at temperatures of 50, 75, 100, and 150 °C for 30 min, respectively. With the increasing RTA temperature, significant agglomeration of the Te film was observed. This behavior is attributed to the reduction in surface energy of Te particles, which drives the migration of Te atoms and the formation of stable inter‐particle bonds, ultimately leading to agglomeration. In contrast, the SEM images in the lower row display the surface of 2D Te layer processed with in situ annealing at 50, 75, 100, and 150 °C during the IPSD process. In situ annealing was carried out by placing the Si wafer or PET substrate on the wafer holder equipped with a heater as shown in Figure 1c. This substrate holder ensures the maintenance of a constant substrate temperature during IPSD process, enabling simultaneous control over both deposition and crystallization processes.

Unlike RTA‐processed Te, the IPSD‐grown Te under in situ annealing exhibited smooth surface morphology and effective crystallization without agglomeration. Consequently, the growth and crystallization of the 2D Te layer is more effectively controlled by in situ annealing, preventing agglomeration across all process temperatures, even at the highest temperature of 150 °C.^[^ ^57^ ^]^ Figure 3b presents the XPS analysis of the IPSD‐grown Te layer using in situ annealing. The XPS survey spectrum displays distinct peaks corresponding to Te3*d*, Te3*p*, and Te4*d*, confirming the successful deposition of Te layer, as seen in Figure S8a (Supporting Information). The presence of these Te 3*d* peaks indicates a well‐defined elemental composition, suggesting that the in situ annealing process effectively maintained the chemical state of Te, minimizing oxidation and ensuring the formation of a high‐quality 2D Te layer. The deconvolution of XPS spectra compares the Te 3*d* region of in situ annealed 2D Te layer with the atomic percentages of each component (Table S2, Supporting Information). In Te 3*d* peak, the Te^0^ peaks are identified at binding energies of 582.6 eV (Te 3*d*_3/2_) and 572.2 eV (Te 3*d*_5/2_), while the Te^4+^ peaks appear at 585.2 eV and 575.1 eV, respectively. The presence of Te^4+^ indicates the formation of a thin layer of tellurium oxide at the interface between the substrate and the Te layer, which is attributed to the oxygen plasma pre‐treatment performed prior to the IPSD process. This thin oxide layer improves the adhesion and chemical stability of the Te layer at the interface.^[^ ^58^, ^59^ ^]^ In contrast, the XPS analysis of Te films subjected to RTA (Figure S8b,c, Supporting Information) shows an increase in the intensity of the Te^0^ peaks, indicating that oxidation occurred not only at the interface but throughout the surface of the Te layer during the RTA process. As expected from SEM results, the RTA process leads to more extensive oxidation across the film surface and agglomeration of Te layer. These findings emphasize that controlled in situ annealing, where a stable tellurium oxide layer forms specifically at the interface, helps maintain the desired properties of the 2D Te layer. Notably, unlike previous works often employing post‐annealing encapsulation methods to suppress oxidation in Te thin films, in situ annealing effectively retains a high ratio of elemental Te (Te^0^) without requiring additional encapsulation. Therefore, in situ annealing is a more effective thermal treatment for growing high‐quality 2D Te layers, as it minimizes unwanted surface oxidation. Figure 3c presents the XRD plots of IPSD‐grown Te layer fabricated with and without in situ annealing. The IPSD‐grown Te layer without in situ annealing shows weak intensities of (100), (101), and (110) peaks due to poor crystallization. In contrast, the in situ annealed Te layer exhibited prominent peaks associated with the (100), (101), and (110) planes, as well as additional (111) and (201) peaks. The significant increase in the intensity of crystalline peaks demonstrates that the in situ annealing process effectively improved the crystallinity of the IPSD‐grown Te 2D layer.^[^ ^60^, ^61^ ^]^ The cross‐sectional TEM image and EDS mapping, as shown in Figure 3d, reveal a uniform distribution of the ≈15 nm thick Te layer deposited on the Si/SiO_2_ substrate. The uniformity of the distribution and the sharpness of the interface of the Te layer indicate that the IPSD process effectively produced a continuous and homogeneous Te film. The uniform distribution and well‐defined interfaces of the Te layer indicate that the IPSD process successfully resulted in a continuous and homogeneous film. The microstructure of the IPSD‐grown Te layer was further analyzed using high‐resolution transmission electron microscopy (HRTEM), as shown in Figure 3e. The HRTEM images reveal distinct interplanar spacings of 0.33, 0.35, and 0.57 nm, which correspond to the (101), (110), and (001) planes of the Te layer, respectively.^[^ ^25^, ^29^, ^33^, ^35^, ^62^, ^63^, ^64^ ^]^ These findings confirm the exceptional crystallinity of the Te layer achieved through the IPSD process. The HRTEM analysis further reveals the uniform arrangement of lattice fringes throughout the Te layer, with no observable grain boundaries or significant defects in the examined regions. The alignment of interplanar spacings with theoretical crystallographic values supports the structural integrity and high quality of the Te layer. Moreover, the incorporation of in situ annealing during the IPSD process plays a critical role in enhancing the crystallinity and sharpening the interface of the Te layer. The absence of amorphous phases and the consistent lattice spacing observed in the HRTEM images indicate that in situ annealing effectively stabilizes the microstructure, minimizing defects and improving the overall quality of the film. Collectively, these results highlight the capability of the IPSD process, combined with in situ annealing, to produce high‐performance Te films suitable for advanced technological applications. These lattice‐resolved images provide visual evidence of the high crystallinity achieved in the IPSD‐grown Te layer through in situ annealing, which is consistent with the XRD findings.

**Figure 4:**
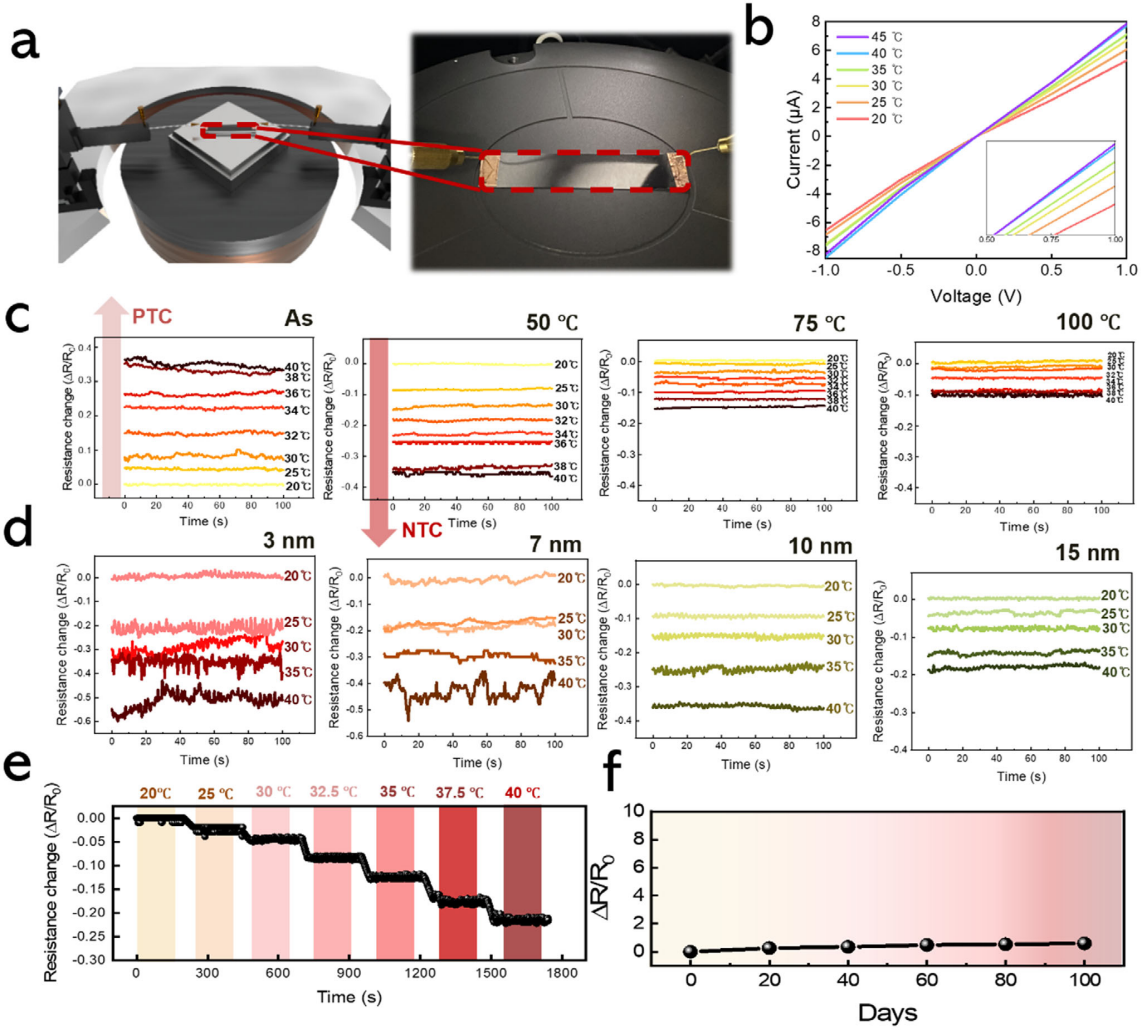
a) Schematic diagram of the test setup and photographic representation of the 2D Te‐based flexible temperature sensor. b) *I–V* characteristics of the 2D Te layer‐based temperature sensor at various temperatures of 20–45 °C. c) Temperature‐dependent resistance change of 2D Te layer‐based temperature sensor grown at different temperatures, showing both positive temperature coefficient (PTC) and negative temperature coefficient (NTC) behaviors. d) Thickness‐dependent temperature response of the 2D Te layer as a function of thickness (3, 7, 10, and 15 nm). e) Continuous resistance response of the 2D Te layer during sequential temperature cycling between 20 and 40 °C. f) Long‐term stability of the 2D Te layer‐based temperature sensor over a 100‐day period.

**Figure** **4**a presents the experimental setup designed to investigate the temperature‐dependent resistance variations of 2D Te layer‐based temperature sensors. The schematic on the left illustrates the sample configuration, where the 2D Te layer is contacted by two electrodes positioned at opposite ends, facilitating precise resistance measurements. On the right, a photograph captures the actual measurement setup within the testing apparatus, with the 2D Te layer sample properly aligned and electrically connected for voltage application. This systematic arrangement enables the evaluation of the sample's electrical properties under controlled temperature conditions. Figure 4b displays the *I–V* characteristics of the 2D Te layer‐based temperature sensors over a temperature range of 20–45 °C. The results demonstrate a clear increase in current with rising temperature, indicative of the negative temperature coefficient (NTC) behavior of the IPSD‐grown 2D Te layer, where electrical resistance decreases as temperature increases. This temperature‐dependent response underscores the suitability of the IPSD‐grown 2D Te layer for high‐temperature environments, positioning it as a promising candidate for temperature sensor applications. Notably, the slope of the *I–V* curves becomes steeper in the 30–40 °C range, signifying enhanced sensitivity in this interval. This characteristic makes the material particularly advantageous for applications requiring precise temperature monitoring, such as human body temperature measurement. Figure 4c compares the temperature‐dependent relative resistance changes for IPSD‐grown 2D Te layers subjected to in situ annealing at 50, 75, and 100 °C, alongside those of the as‐deposited Te layer. The as‐deposited Te layer sensor exhibits positive temperature coefficient (PTC) behavior, where resistance increases with rising temperature. In contrast, the 2D Te layers annealed at elevated temperatures of 50, 75, and 100 °C display NTC behavior, characterized by a decrease in resistance as temperature increases. Without in situ annealing, the IPSD‐grown Te layer exhibits an amorphous or partially crystallized structure, characterized by localized defects that hinder charge transport. The poor conductivity of the amorphous Te layer, where electron mobility decreases with increasing temperature, results in PTC behavior. In contrast, in situ annealing significantly enhances the crystallinity of the 2D Te layer, facilitating grain growth and reducing grain boundary density. This improved crystallinity of the 2D Te facilitates charge transport by simplifying conductive pathways and enhancing electron mobility. As a result, thermally activated electrons can traverse these conductive paths more efficiently, leading to the observed NTC behavior. Furthermore, the intrinsically low lattice thermal conductivity of Te enhances charge transport efficiency at elevated temperatures.^[^ ^65^ ^]^ The observed NTC behavior in the in situ annealed Te layer can be accurately modeled using the fundamental NTC equation, providing a theoretical framework to describe the temperature‐dependent resistance relationship.^[^ ^66^ ^]^

(1) $$R_{T}=R_{T0}\cdot exp\left(B\left(\frac{1}{T}-\frac{1}{T_{0}}\right)\right)\;$$

In this equation, *R_T_* represents the resistance at temperature *T*, while *R*_*T*0_ denotes the resistance at the reference temperature *T_0_*. The material‐specific B coefficient quantifies the sensitivity of resistance to temperature changes, with higher values indicating a stronger response to temperature variations. As temperature *T* increases, the exponential term $exp(-\frac{B}{T})$ decreases, leading to a reduction in *R_T_*. This inverse relationship is characteristic of NTC behavior, wherein elevated temperatures enhance electron mobility, thereby increasing conductivity and reducing resistance. The observed NTC response in the in situ annealed 2D Te layer can also be attributed to the Seebeck effect, which signifies increased electron mobility with rising temperature, further contributing to the decrease in resistance. This model effectively captures the temperature sensitivity of the IPSD‐grown Te layer, especially within the range of 50–100 °C. Notably, the IPSD‐grown Te layer in situ annealed at 50 °C exhibited a relative resistance change from 0.0 to ‐0.4, demonstrating exceptionally high‐temperature sensitivity. However, the sensitivity was approximately halved for the layer annealed at 75 °C. Figure 4d shows the relative resistance changes of IPSD‐grown Te layers with varying thicknesses of 3, 7, 10, and 15 nm, measured across a temperature range of 20 to 40 °C. The 3 and 7 nm thick Te layers exhibited high sensitivity to temperature variations but displayed unstable electrical characteristics, with a sharp increase in resistance observed above 35 °C. In contrast, Te layers with thicknesses exceeding 10 nm demonstrated stable resistance variations across the entire temperature range, maintaining consistent behavior even beyond 35 °C. Among these, the 10 nm thick film exhibited an optimal balance between thermal stability and temperature sensitivity, making it the most suitable choice for reliable sensing applications. Figure 4e shows the resistance change of the IPSD‐grown Te layer‐based temperature sensor during continuous temperature variation from 20 to 40 °C. A stable resistance response was recorded throughout the temperature range, with a gradual decrease in relative resistance change as the temperature increased. This trend confirms the high sensitivity of the Te thin film to temperature fluctuations, allowing for robust assessment of its temperature responsiveness over a broad range. Additionally, Te's inherent resistance to oxidation contributes to the long‐term stability of the wearable temperature sensor, ensuring reliable performance in extended operational conditions. The device also maintained stable response characteristics under repeated thermal cycling. As shown in Figure S9 (Supporting Information), ten consecutive sensing cycles between 20 and 40 °C were conducted, with each cycle consisting of a 1000 s period of heating and passive cooling. While the temperature did not fully return to baseline within each cycle, the resistance response remained consistent without significant drift. The maximum resistance change (ΔR/R₀) decreased slightly from 0.387 in the first cycle to 0.364 in the tenth, indicating a sensitivity drop of ≈6%, and confirming robust operational reproducibility. This strong oxidation resistance ensures that the temperature sensor retains its performance without significant degradation or surface deterioration over extended periods. To verify this, the resistance change of the sensor was monitored at 10‐day intervals over a period of 100 days. As shown in Figure 4f, the relative resistance changes between the initial measurement (Day 0) and after 100 days was less than 1%, demonstrating the exceptional long‐term stability of the IPSD‐grown Te layer‐based temperature sensor. To further evaluate the environmental stability of the sensor, an accelerated aging test was conducted at 85 °C and 85% RH for 2 h. As shown in Figure S10 (Supporting Information), the temperature‐dependent resistance response remained stable within ≈2% fluctuation across 20–40 °C, confirming that the sensor maintains reliable performance even after exposure to harsh conditions.

**Figure 5:**
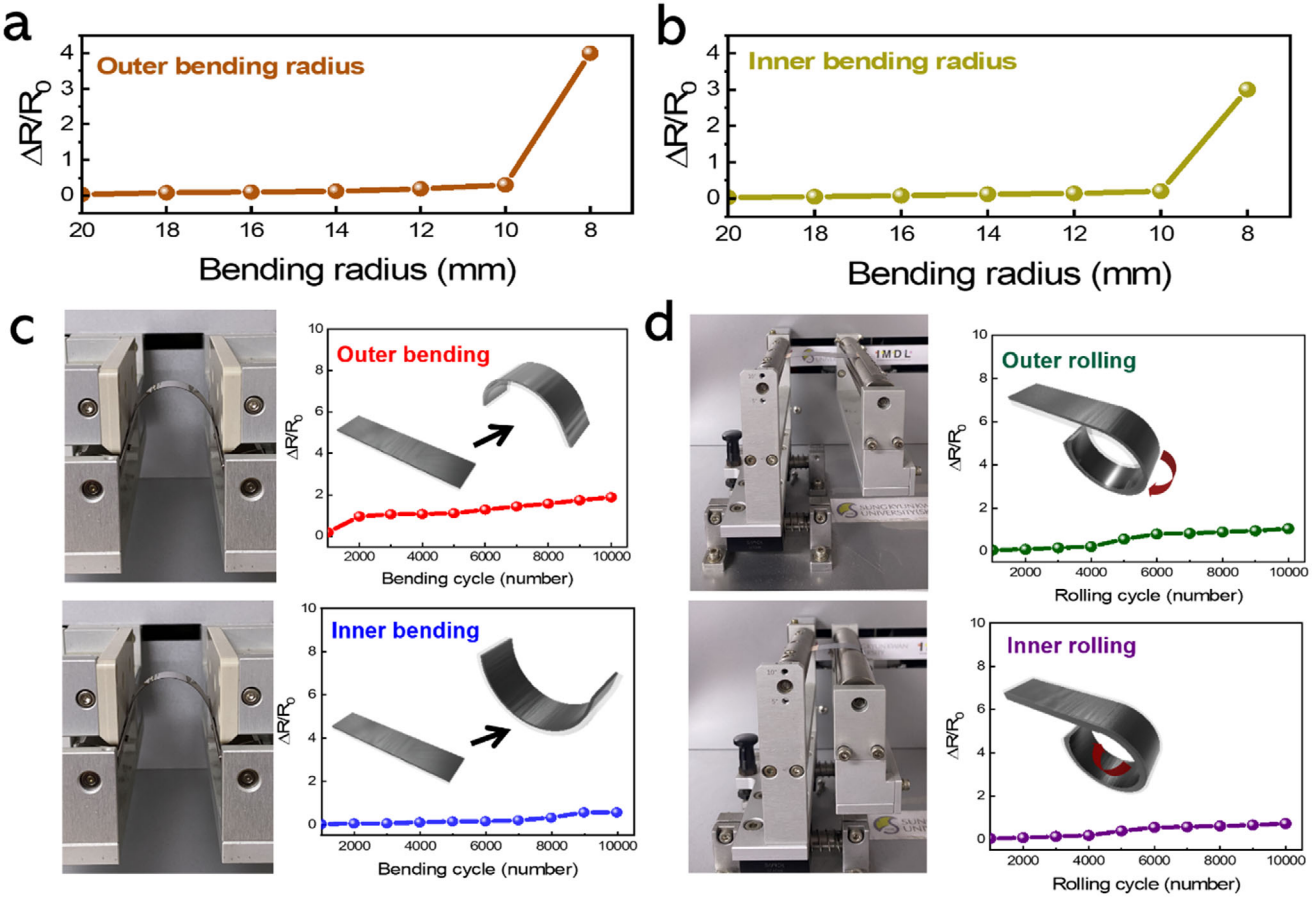
Relative resistance change (∆R/R_0_) of IPSD‐grown Te layer as a function of bending radius under a) outer and b) inner bending conditions. c) Photograph and results of bending durability test showing resistance change over repeated 10 000 cycles for both outer and inner bending. d) Rolling durability test results indicating resistance stability after 10 000 repeated cycles for both outer and inner rolling conditions.

In this work, the in situ annealing of IPSD‐grown 2D Te layer was conducted at a relatively low temperature of 50 °C. This controlled approach was essential for preserving the mechanical flexibility of the Te layer while considering the transition temperature (75 °C) of the PET substrate.^[^ ^67^ ^]^ Higher annealing temperatures are known to promote excessive grain growth and the formation of rigid crystalline structures, which can compromise flexibility by increasing brittleness. In contrast, the moderate annealing temperature employed in this study effectively mitigated these effects, achieving an optimal balance between crystallinity and mechanical durability. The flexibility and durability of the IPSD‐grown 2D Te layer were further evaluated through extensive bending and rolling tests, as detailed in **Figure** **5**. Initially, the outer and inner bending radius limits of the Te layer were determined using a lab‐designed bending tester, as shown Figure 5a,b. The relative resistance change (ΔR/R₀) was measured for both outer and inner bending radii, revealing minimal variation for radii greater than 10 mm. However, a sharp increase in resistance was observed at a radius of 8 mm, indicating the mechanical flexibility limit of the IPSD‐grown Te layer. Subsequently, long‐term durability tests were performed, with 10 000 bending cycles conducted under both outer and inner bending conditions (Figure 5c). The results indicated a resistance change of less than 4% for outer bending and 2% for inner bending, demonstrating high stability under repeated mechanical stress. Similarly, the rolling tests (Figure 5d) confirmed excellent durability, with ΔR/R₀ maintained below 3% for outer rolling and 2% for inner rolling throughout 10 000 cycles. The mechanical stability recorded in these tests suggests that the microstructure of the Te thin film remains largely intact during bending and rolling, preventing significant disruption of conductive pathways. Notably, the ΔR/R₀ values remained below 4% even after extensive cyclic testing, underscoring the exceptional mechanical flexibility and durability of the IPSD‐grown Te layer. To further verify that the sensing functionality was not compromised by mechanical stress, we evaluated the temperature response of the sensor after 10 000 cycles of bending and rolling. As shown in Figure S11 (Supporting Information), the sensor maintained clear, stable resistance changes across 20–40 °C under all deformation modes. The bending radius tests provided crucial insights into the mechanical flexibility limits, which informed the subsequent cyclic durability evaluations. The lower resistance changes detected under inner bending and rolling conditions can be attributed to reduced tensile stress on the film surface, emphasizing the importance of deformation mode in flexible electronic applications. Overall, the comprehensive bending and rolling tests confirmed the robustness of the IPSD‐fabricated Te thin films under extensive mechanical deformation. The minimal resistance change recorded across 10 000 cycles highlights the film's durability, making it a promising candidate for reliable integration into flexible electronic applications.

## 3 Conclusion

We successfully developed high‐quality flexible Te thin films using an optimized IPSD process, in combination with in situ annealing and O₂ plasma pre‐treatment. This advanced approach facilitates the fabrication of highly sensitive and reliable temperature sensors for next‐generation wearable and flexible electronic devices. The IPSD process effectively addressed common challenges associated with plasma damage and surface defects by preventing direct exposure of the substrate to plasma, thereby preserving the unique helical chain structure of Te and ensuring uniform thin film deposition. Additionally, O₂ plasma pre‐treatment enhanced the surface energy of the substrate by removing organic contaminants and impurities, significantly improving the adhesion of the Te thin films. The in situ annealing process further stabilized the crystalline structure during deposition, eliminating the need for additional thermal treatments.

The optimized Te thin films exhibited excellent electrical and mechanical properties, achieving a high carrier mobility of 103 cm^2^ V^−1^ s^−1^ and a low surface roughness of 0.778 nm. These films demonstrated stable Negative Temperature Coefficient (NTC) behavior, which is essential for temperature sensing applications. Notably, Te thin films treated with in situ annealing demonstrated high sensitivity and rapid response across the human body temperature range (20–40 °C), making them highly suitable for precise temperature monitoring. Mechanical durability tests, including 10 000 cycles of bending and rolling, confirmed the robustness of the Te thin films, with a minimal resistance change of less than 4%, showcasing exceptional flexibility and resilience. Furthermore, long‐term stability tests conducted over a period of 100 days revealed a resistance change of less than 1%, underscoring the enhanced reliability of the Te thin films, attributed to the combined effects of O₂ plasma pre‐treatment and in situ annealing, which contribute to both the mechanical durability and long‐term stability of the films.

In conclusion, the synergistic combination of the IPSD process, O₂ plasma pre‐treatment, and in situ annealing facilitated the production of Te thin films with high carrier mobility, superior mechanical flexibility, and long‐term stability. These findings demonstrate that the optimized thin films are ideal candidates for integration into next‐generation wearable and flexible electronic devices. The unique capabilities of the IPSD process, coupled with the advantageous properties of Te, overcome the limitations of conventional deposition methods, paving the way for scalable, high‐performance thin‐film sensor applications.

## 4 Experimental Section

### Deposition and Preparation Conditions for Optimized Te Thin Films

Te thin films were deposited using the IPSD system. The Te target, with 99.99% purity (iTASCO), measured 505 mm × 86 mm × 6 mm (thickness) and consisted of five tiles, each measuring 101 mm × 86 mm × 6 mm. Two targets for deposition were facing each other to generate plasma. The substrates used were a 6‐inch Si/SiO_2_ (200 nm) wafer and a PET film with dimensions of 20 mm × 80 mm. The Si/SiO_2_ substrate was cleaned using an ultrasonic cleaner, with sequential 10‐min treatments in acetone, DI water, and isopropyl alcohol. After cleaning, the substrate was dried in an oven at 80 °C for 1 h to remove any remaining residue. The PET substrate was supplied with a protective cover on the deposition surface, which was removed just before use, eliminating the need for additional cleaning. Prior to placing the substrates in the chamber, an O_2_ plasma surface treatment was applied to enhance surface adhesion. During this process, high‐purity oxygen gas (99.99%) was supplied at a flow rate of 10 sccm, with an RF power of 200 W applied for 240 s. The plasma treatment contributed to the removal of organic contaminants and activated the substrate surface. To ensure the reproducibility of the experiment, the base pressure was maintained at 9.0 × 10^−7^ Torr, with both the target‐to‐target distance and a target‐to‐substrate distance set at 6 cm. Plasma conditions were optimized using a DC power of 50 W, a working pressure of 2.0 mTorr, and an argon gas flow rate of 40 sccm. Additionally, the heater temperature was set to 50 °C to maintain the temperature stability of the substrate during deposition. For comparison with in situ annealing, rapid thermal annealing (RTA) was performed at 100 °C under a vacuum of 9.9 × 10^−4^ Torr for 30 min.

### Structural, Electrical and Mechanical Analysis of Te Thin Films

To analyze the crystallinity and structural properties of the Te thin films, a Raman spectrometer (Witec, Alpha 300M+) with a 532 nm excitation laser was utilized to observe the characteristic vibrational modes of Te. The charge carrier mobility of the thin films was assessed using a Hall effect measurement system (Ecopia, HMS‐4000AM), while a UV–vis spectrometer (Jasco, UV‐670) was employed to evaluate their optical properties. The surface morphology of the thin films was analyzed using field emission scanning electron microscopy (FESEM; JEOL, JSM‐7600F), and atomic force microscopy (AFM; Park Systems, NX‐10) was employed to assess the impact of O₂ plasma surface treatment on topography. Additionally, high‐resolution X‐ray diffraction (HR‐XRD; Rigaku, SmartLab) was employed to investigate variations in crystallinity with and without annealing of the thin films. The microstructure and chemical composition of the Te thin films were analyzed using high‐resolution transmission electron microscopy (HR‐TEM; JEOL, JEM‐2100F), which also enabled scanning transmission electron microscopy (STEM) and energy‐dispersive X‐ray spectroscopy (EDS) to investigate atomic‐level grain boundaries, interfacial characteristics, and elemental distribution. Furthermore, X‐ray photoelectron spectroscopy (XPS; ThermoFisher Scientific, K‐alpha) was employed to investigate changes in chemical composition under different annealing methods, focusing on the relative shifts in Te⁰ and Te⁴⁺ peaks. To assess the mechanical stability of the thin films, laboratory‐designed bending and rolling equipment was utilized. The mechanical properties were evaluated by monitoring resistance changes after 10 000 cycles under inner and outer radius conditions.

### Fabrication and Analysis of Wearable Te Temperature Sensor

To use the optimized Te thin film as a temperature sensor, the film was placed on a PET substrate, with Mo electrodes (20 mm × 5 mm) deposited at each end using a four‐inch magnetron sputtering process. The Mo target (DASOMRMS) was deposited under the following conditions: a base pressure = 9.9 × 10^−7^ Torr, DC power = 100 W, working pressure = 3 mTorr, and argon gas flow rate = 20 sccm, resulting in a Mo thickness of 30 nm. To ensure reliable electrical connectivity and minimize contact resistance, copper tape (20 mm × 5 mm) was applied over the Mo electrodes, providing stable electrical contacts for precise measurements. The fabricated temperature sensor was tested for resistance changes in response to temperature fluctuations using a 2‐terminal probe station (OTS Technology, MST‐4000A) in combination with a hot chuck controller (MSTECH, MST‐1000H). The performance characteristics of the Te thin film‐based temperature sensor were evaluated by analyzing the resistance changes with temperature. Semiconductor properties were measured with a semiconductor parameter analyzer (Tektronix, Keithley 4200‐SCS).

## Conflict of Interest

The authors declare no conflict of interest.

## Supporting information



Supporting Information

Supplemental Movie 1

## Data Availability

The data that support the findings of this study are available from the corresponding author upon reasonable request.
