# Supplementary material for: High‐Performance Flexible 2D Tellurium Semiconductor Grown by Isolated Plasma Soft Deposition for Wearable and Flexible Temperature Sensors
Source: Small Methods. 2025 Jun 29;9(8):2500379. doi: 10.1002/smtd.202500379 (PMC12391622; doi:10.1002/smtd.202500379)
Supplement: Supplementary file 1 — Supporting Information [file SMTD-9-2500379-s002.docx]

Supporting Information

**High-Performance Flexible 2D Tellurium Semiconductor Grown by Isolated Plasma Soft Deposition for Wearable and Flexible Temperature Sensors**

*Tae-Yang Choi, Jun-Hyeok Kang, Jong-Hyun Jang and Han-Ki Kim**

T. -Y. Choi, J. -H. Kang, J. -H. Jang, Prof. H.-K. Kim

School of Advanced Materials Science & Engineering, Sungkyunkwan University (SKKU), 2066 Seobu-ro, Jangan-gu, Suwon, Gyeonggi-do 16419, Republic of Korea

Email: hankikim@skku.edu


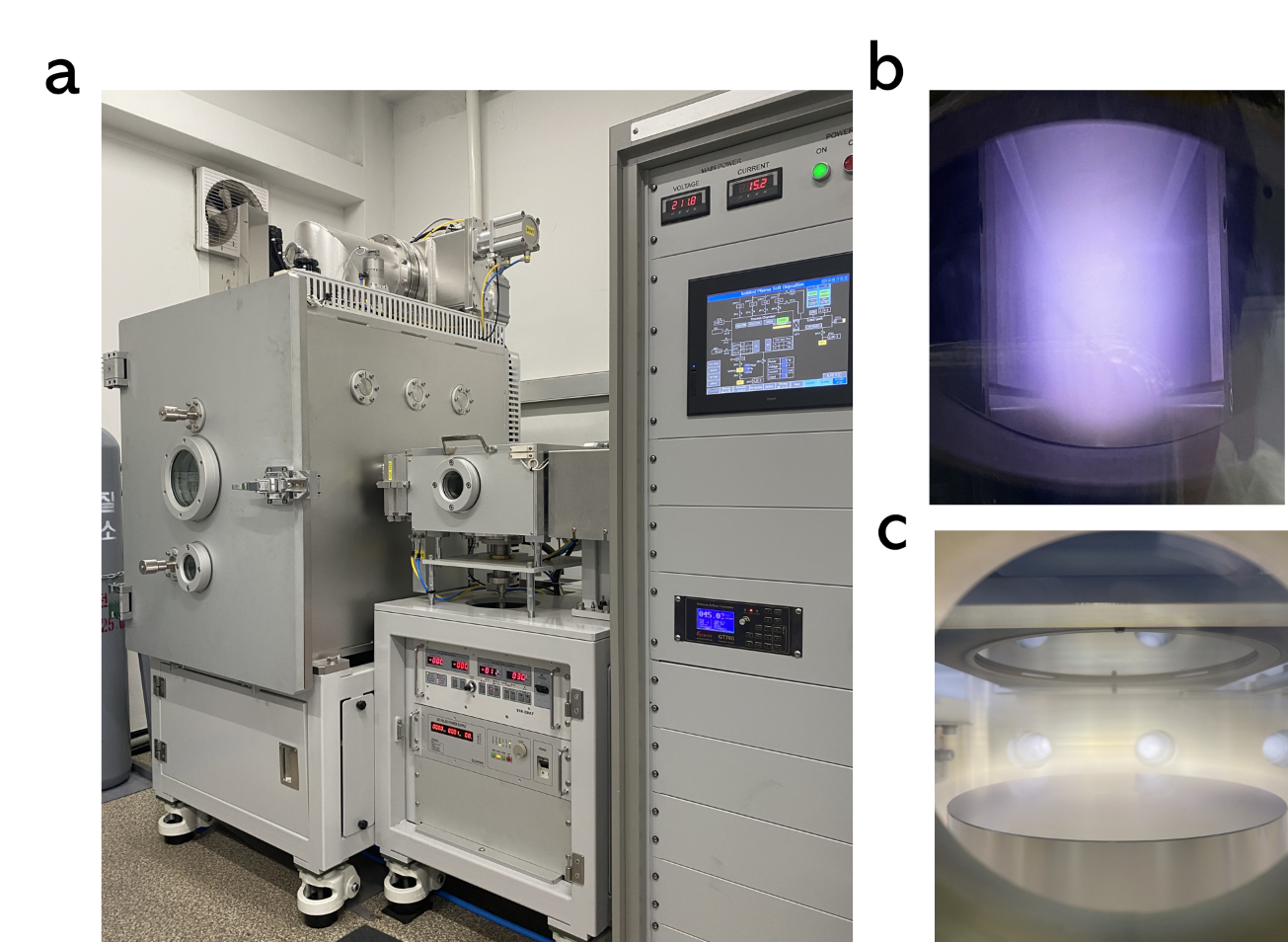


**Figure S1.** a) Photograph of the isolated plasma soft deposition (IPSD) system used for Te thin film fabrication. b) Photograph of high-density plasma confined between facing Te targets during IPSD process. c) Photograph of O_2_ plasma used for the pre-treatment of surface Si or PET substrates.

**Figure S1** illustrates O_2_ plasma surface pre-treatment process and its role in the deposition of 2D Te thin films, 15 nm in thickness, characterized via Raman spectroscopy. As shown in **Figure S1a**, the O₂ plasma treatment effectively removes surface organic compounds and impurities, by converting them into volatile by-products such as H_2_O and CO_2_. This process not only cleans the substrate surface but also increase its surface energy, as depicted in **Figure S1b**, thereby improving adhesion for subsequent Te deposition. The selection of O_2_ as the plasma gas, as shown in **Figure S1c**, was driven by its superior chemical reactivity, which facilitates more efficient contaminant removal compared to the physical sputtering effects of Ar or N_2_ plasma.^[S1]^ The enhanced cleaning efficiency of O₂ plasma ensured that the Te thin film was deposited on a cleaner and more chemically reactive substrate, thereby minimizing defects that could compromise film performance.


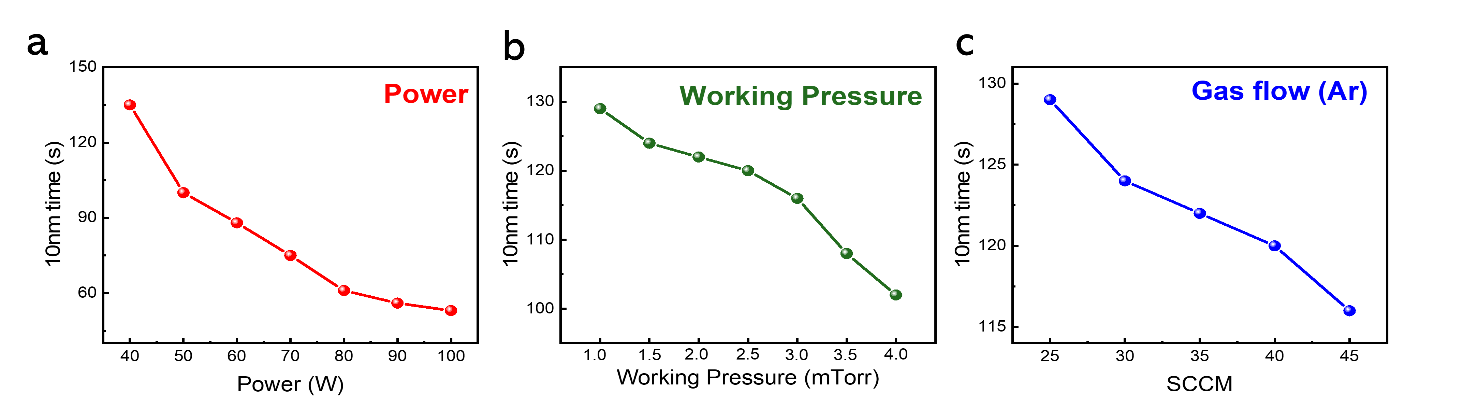


**Figure S2.** Deposition rate analysis showing the time required to achieve a 10 nm thickness as a function of a) DC power, b) working pressure, and c) Ar gas flow.


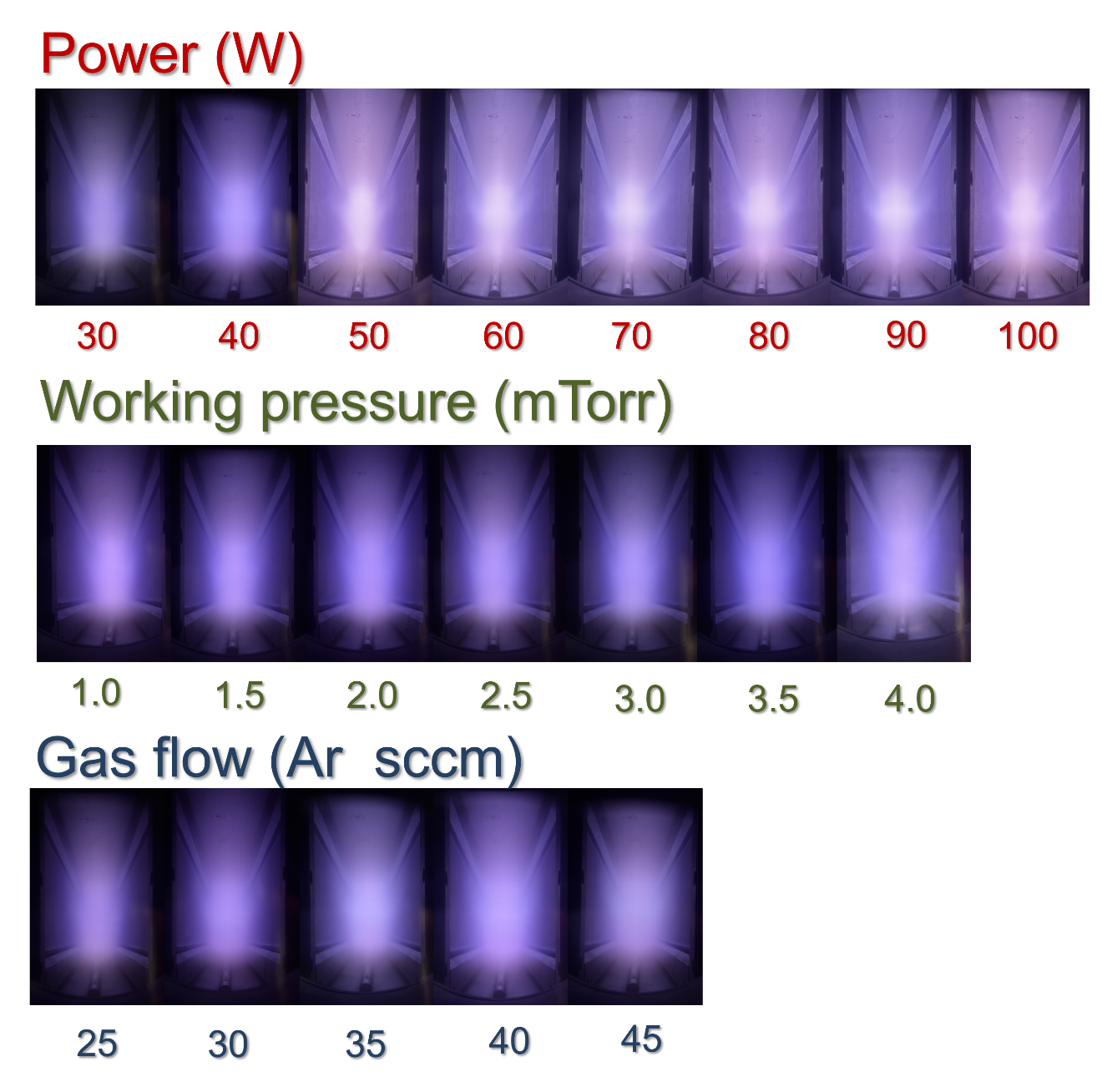


**Figure S3.** Photographs of plasma illustrating the effect of process parameters across a wide range: Power (30–100 W), Working Pressure (1.0–4.0 mTorr), and Gas Flow (25–45 sccm).


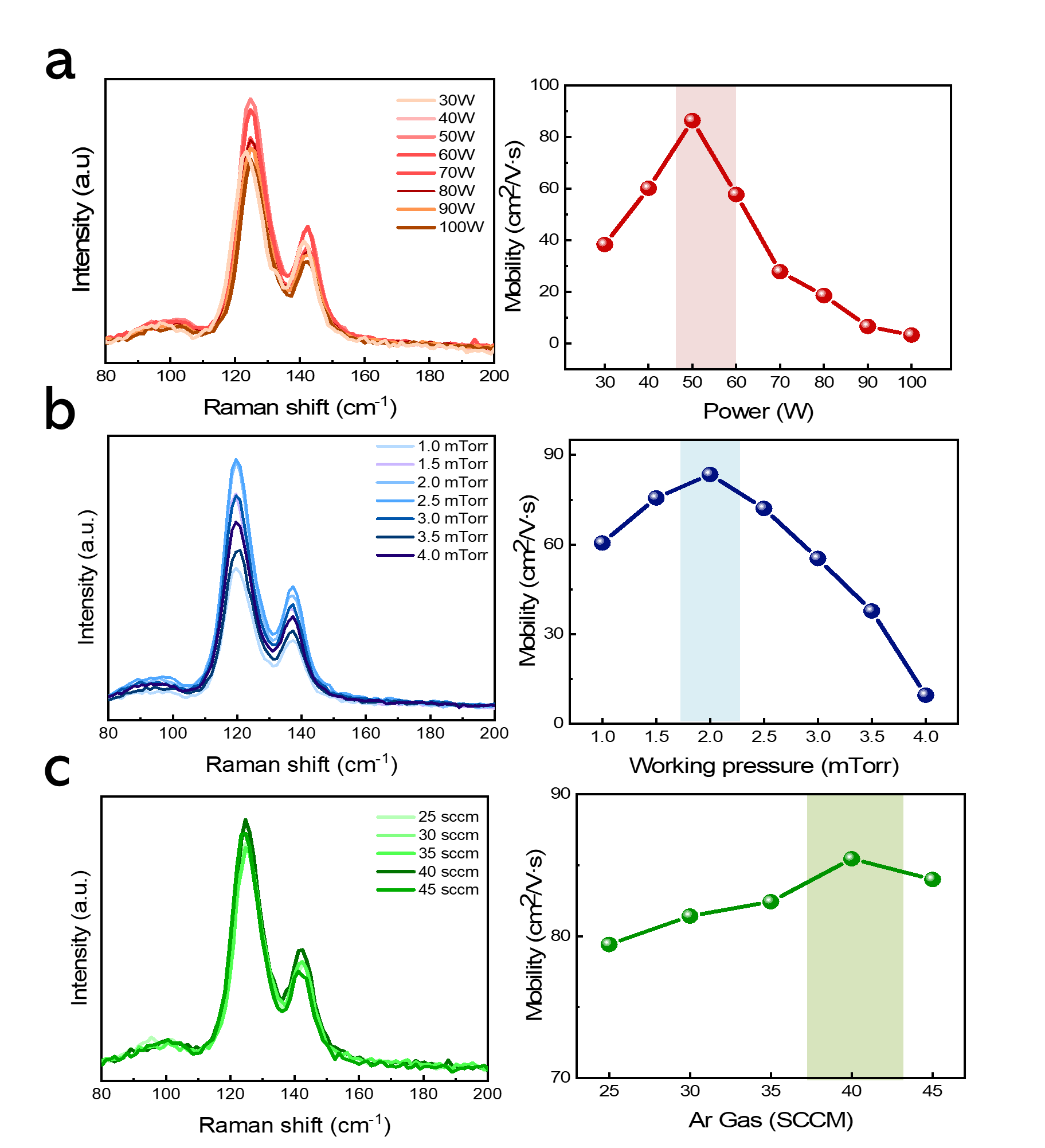


**Figure S4.** Raman analysis and mobility trends of Te thin films under varying conditions: a) power (30–100 W), b) working pressure (1.0–4.0 mTorr), and c) gas flow (25–45 sccm). The left panels present the Raman spectra, while the right panels show the corresponding mobility measurements.

**Figure S4** presents the Raman spectra and mobility results of Te thin films synthesized under varying conditions of power, working pressure, and Ar gas flow in the IPSD system. The characteristic Raman peaks of the 2D Te layer, corresponding to the E₁ mode (93 cm⁻¹), A₁ mode (119 cm⁻¹), and E₂ mode (140 cm⁻¹), were consistently observed across all experimental conditions, underscoring the robustness and reproducibility of the IPSD growth process.^[S2]^ **Figure S4a** shows that the maximum Raman intensity was achieved at a DC power of 50 W, corresponding to the highest mobility of 88 cm²/V·s. At an optimal working pressure of 2.0 mTorr, as depicted in **Figure S4b**, a mobility of 87.4 cm²/V·s was recorded. Furthermore, **Figure S4c** reveals that adjusting the gas flow rate to 40 sccm resulted in a mobility of 86.4 cm²/V·s. These findings underscore the strong correlation between IPSD growth parameters and the electrical properties of the Te thin films, highlighting the ability to fine-tune deposition conditions for enhanced performance.


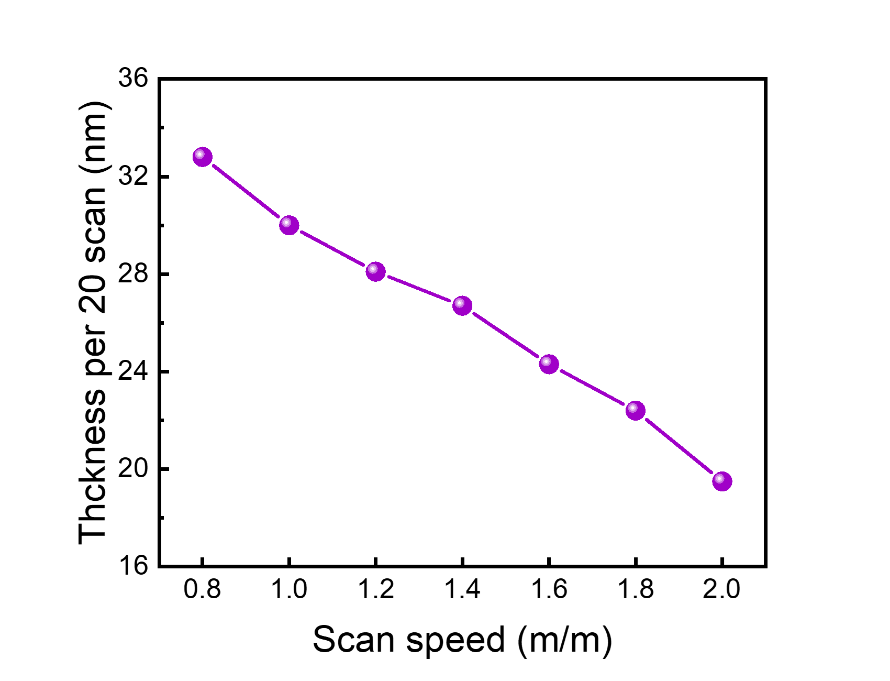


**Figure S5.** Thickness of Te thin films as a function of scan speed over 20 scan cycles.


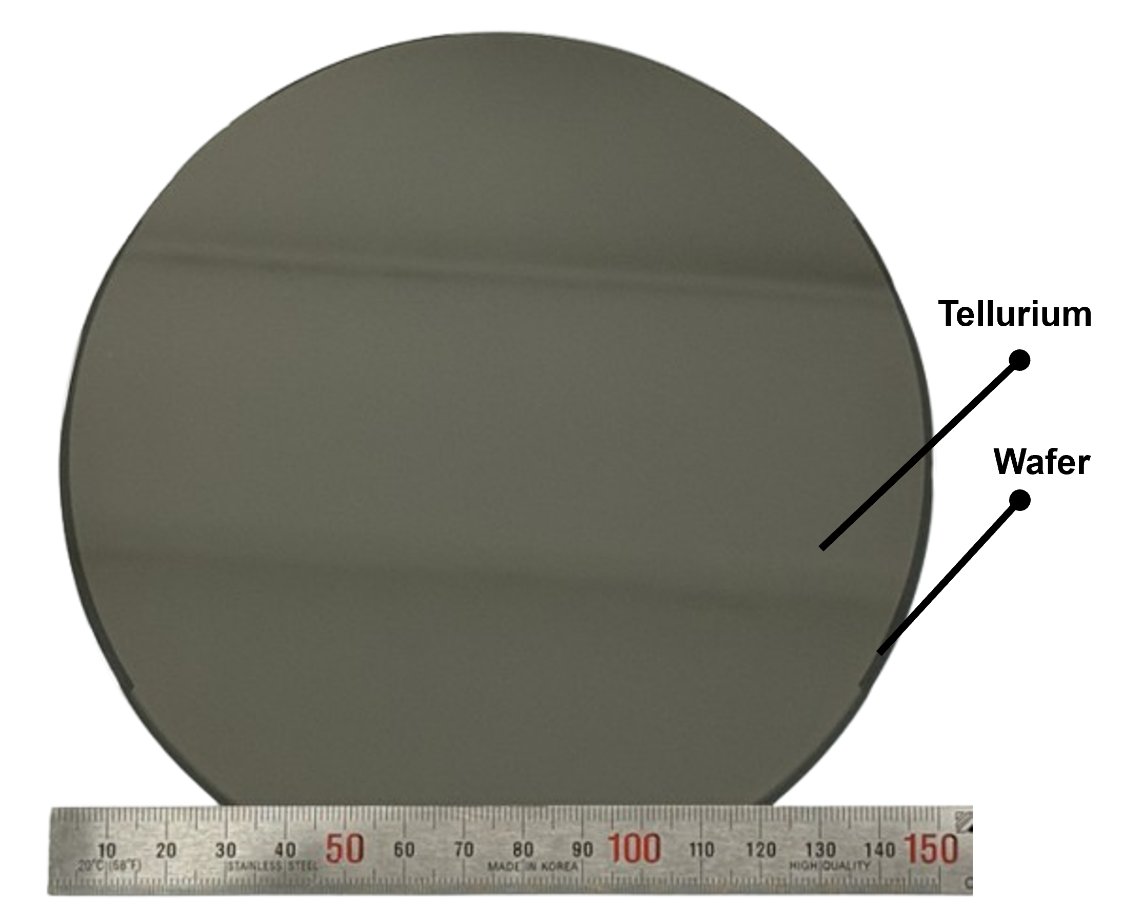


**Figure S6.** Photograph of a 6-inch wafer demonstrating the uniform Te thin film deposition


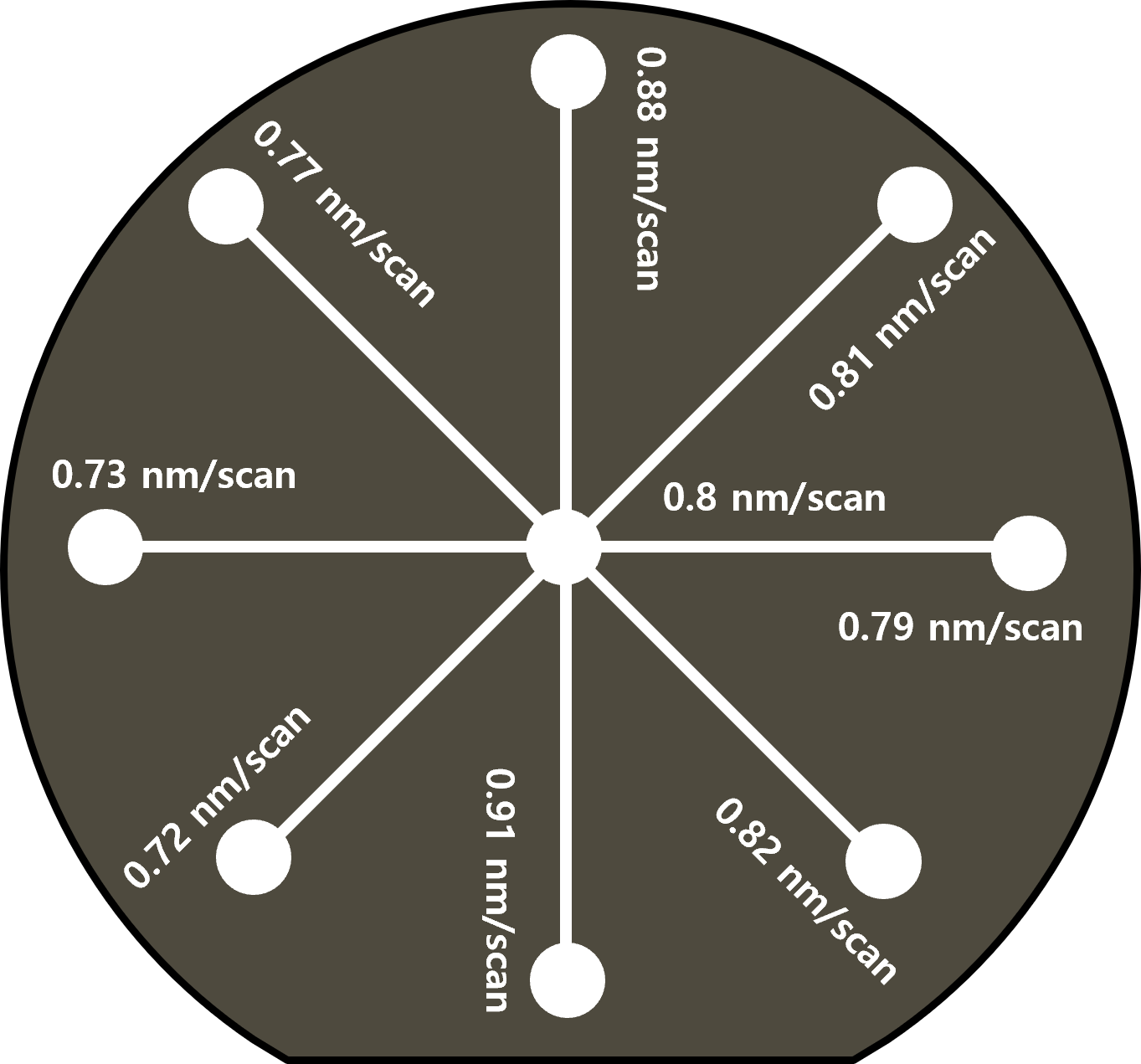


**Figure S7.** Thickness distribution map of Te thin films at a scan speed of 2.0 mm/s, showing the nm/scan values across different substrate positions.

Mean $=\frac{\sum Values}{n}$ = $\frac{0.77+0.88+0.81+0.80+0.79+0.82+0.91+0.72+0.73}{9}$ = 0.803 nm/scan (1)

$Standard Deviation= \sqrt{\frac{\sum{(Value-Mean)}^{2}}{n}}$= 0.0589 nm/scan (2)

$Error Rate \left( \% \right)= \frac{Standard Deviation}{Mean} \times100$ = $\frac{0.0589}{0.0803} \times100=7.3\%$ (3)


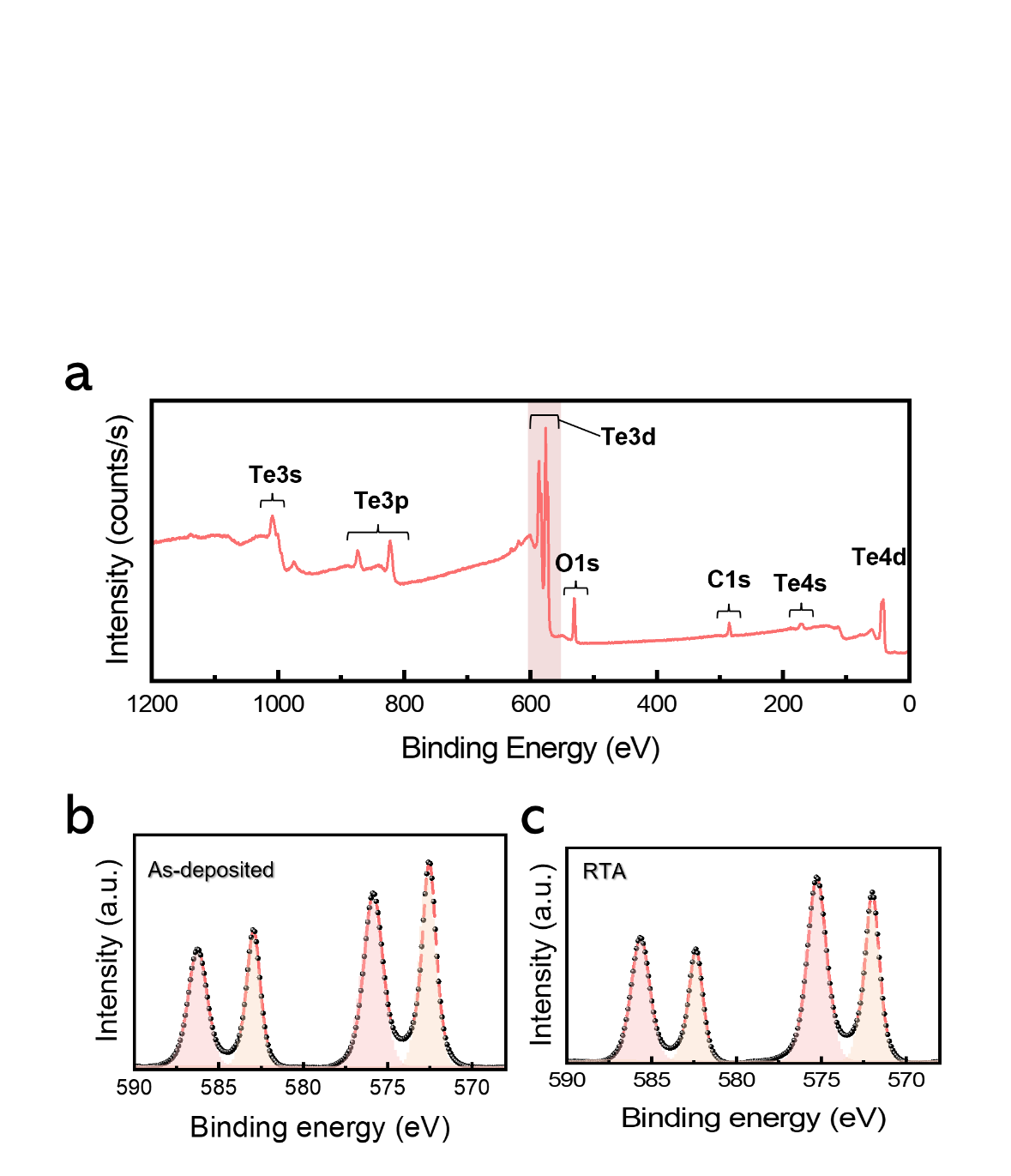


**Figure S8.** a) XPS survery spectrum of *in-situ* annealed Te thin films. b) XPS spectra of unannealed Te thin films. c) XPS spectra after rapid thermal annealing (RTA), illustrating changes in chemical states.


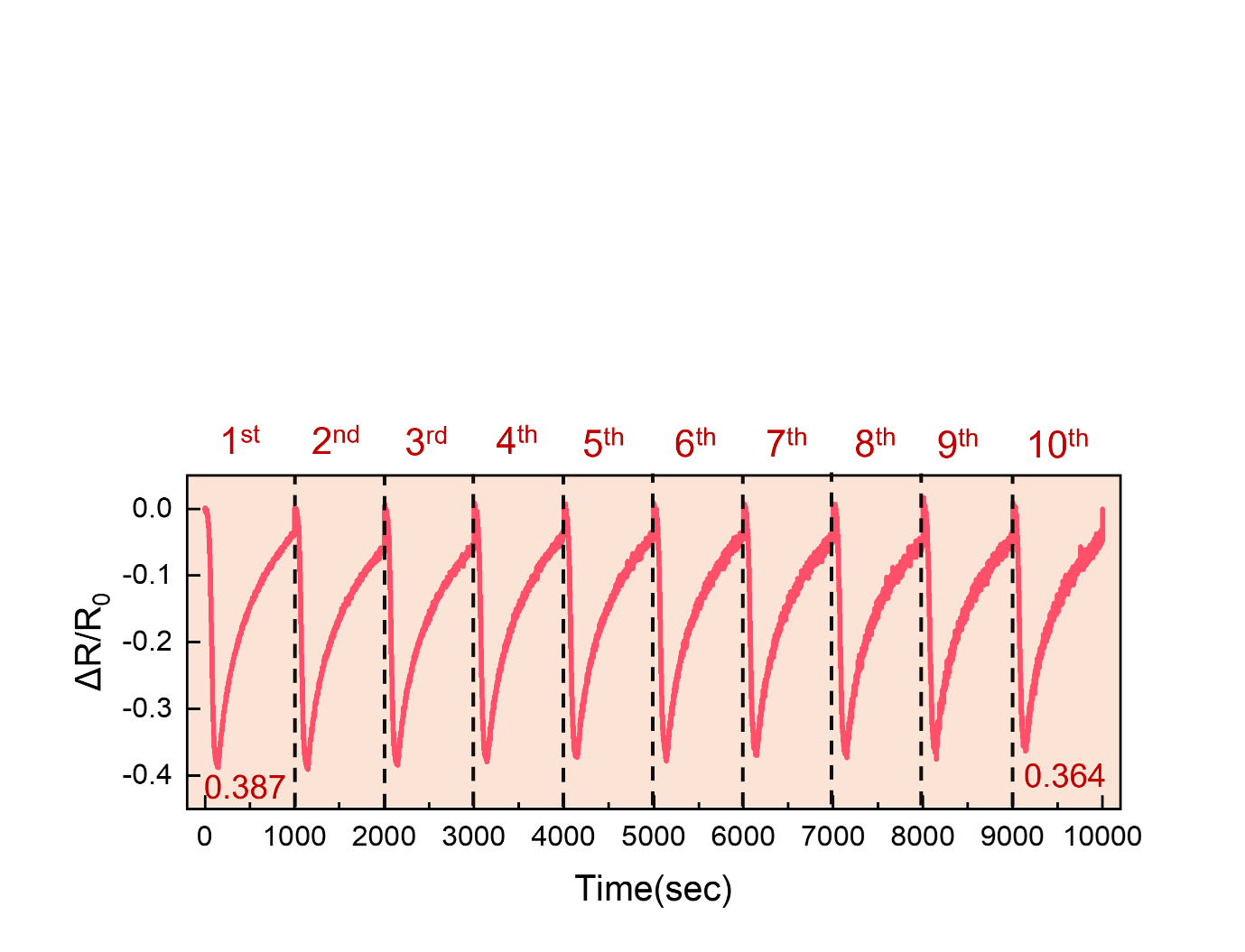


**Figure S9.** Resistance response of the 2D Te-based temperature sensor measured over ten repeated sensing cycles.


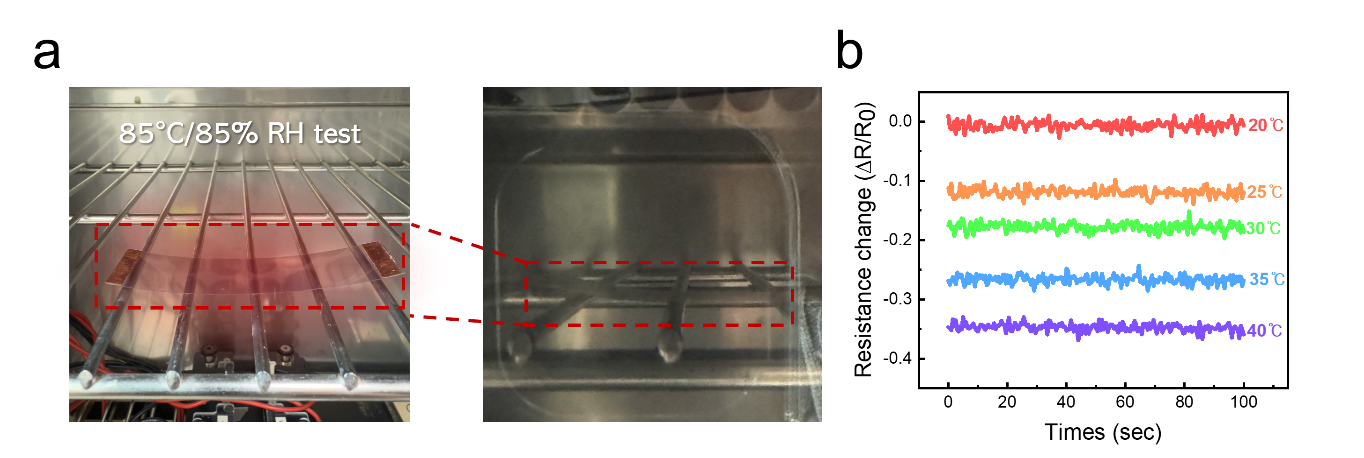


**Figure. S10.** a) Photograph of the temperature sensor subjected to the 85 °C/85% RH test. b) Performance evaluation of the temperature sensor at various temperatures (20–40 °C) after the 85 °C/85% RH test.


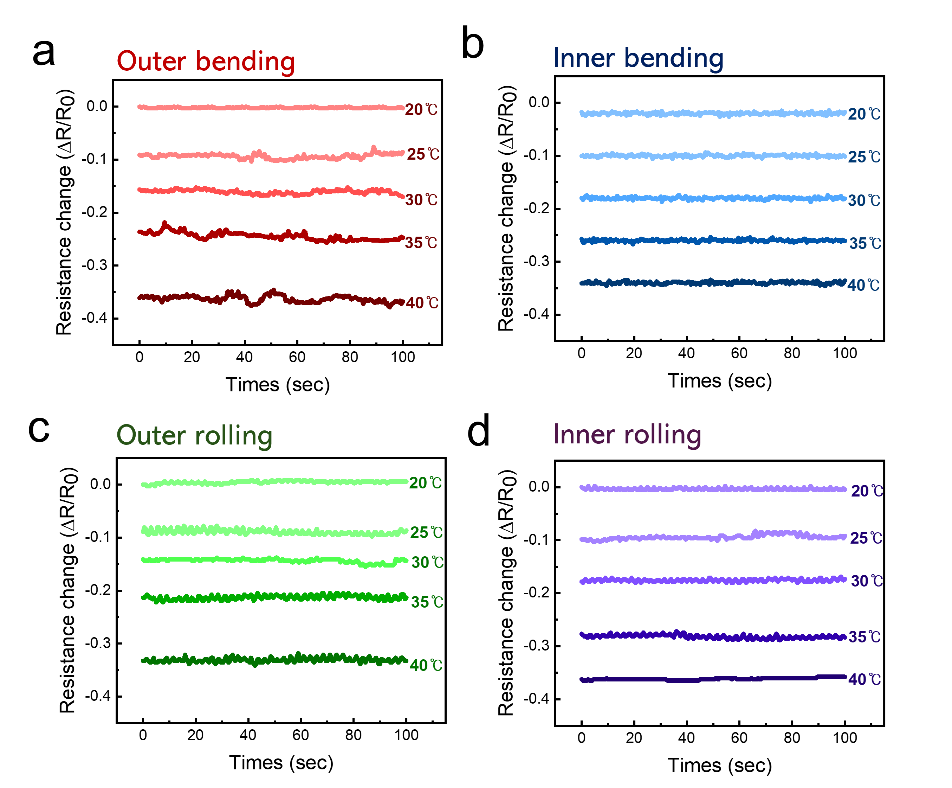


**Figure S11.** Temperature-dependent resistance changes of the 2D Te-based flexible temperature sensor measured at different temperatures (20–40 °C) after 10,000 cycles a) outer bending. b) outer rolling. c) inner bending. d) inner rolling.

**Table S1.** Deposition rates of Te thin films grown using the IPSD system under varying DC power (30–100 W), working pressure (1.0–4.0 mTorr), and Ar gas flow (25–45 sccm).

| Power  (W) | Working pressure  (mTorr) | Ar  (sccm) | Deposition rate  (nm/s) |
| --- | --- | --- | --- |
| **30** | 2.5 | 30 | 0.0667 |
| **40** | 2.5 | 30 | 0.0741 |
| **50** | 2.5 | 30 | 0.1 |
| **60** | 2.5 | 30 | 0.1136 |
| **70** | 2.5 | 30 | 0.1333 |
| **80** | 2.5 | 30 | 0.1639 |
| **90** | 2.5 | 30 | 0.1786 |
| **100** | 2.5 | 30 | 0.1887 |
| 50 | **1.0** | 30 | 0.0795 |
| 50 | **1.5** | 30 | 0.082 |
| 50 | **2.0** | 30 | 0.08441 |
| 50 | **2.5** | 30 | 0.0882 |
| 50 | **3.0** | 30 | 0.0903 |
| 50 | **3.5** | 30 | 0.0932 |
| 50 | **4.0** | 30 | 0.095 |
| 50 | 2 | **25** | 0.0775 |
| 50 | 2 | **30** | 0.0806 |
| 50 | 2 | **35** | 0.082 |
| 50 | 2 | **40** | 0.0842 |
| 50 | 2 | **45** | 0.0879 |

**Table S2.** XPS data of Te thin films under various annealing conditions, displaying atomic percentages (at%) of Te (IV) and Te components in 3d₃/₂ and 3d₅/₂ states for as-deposited, RTA, and *in-situ* annealed samples.

| **Annealing condition** | ***Te^4+^* 3*d*_3/2_**  **(at%)** | ***Te^0^* 3*d*_3/2_**  **(at%)** | ***Te^4+^* 3*d*_5/2_**  **(at%)** | ***Te^0^* 3*d*_5/2_**  **(at%)** |
| --- | --- | --- | --- | --- |
| As-deposited | 18.7 | 21.5 | 27.3 | 32.5 |
| RTA | 22.2 | 17.2 | 33.1 | 27.5 |
| *In-situ* annealing | 9.8 | 28.3 | 22.8 | 39.1 |

**Reference**

[S1] N. Recek, A. Vesel, *Mater. Tehnol.* **2014**, *48*, 893.

[S2] J. W. Liu, F. Chen, M. Zhang, H. Qi, C. L. Zhang, S. H. Yu, *Langmuir* **2010**, *26*, 11372.
